# Radiation therapy after radical surgery in prostate cancer

**DOI:** 10.3332/ecancer.2023.1565

**Published:** 2023-06-27

**Authors:** Roberto Paz-Manrique, Gerard Morton, Fernando Quiroa Vera, Santiago Paz-Manrique, Andrés Espinoza-Briones, Carlos Morante Deza

**Affiliations:** 1 Oncosalud – AUNA, Lima 15036, Peru; 2 Department of Radiation Oncology, Sunnybrook Odette Cancer Center, Toronto, ON M4N 3M5, Canada; 3 Instituto Nacional de Enfermedades Neoplásicas (INEN), Lima 15038, Peru; 4 Universidad Católica de Santa Maria, Arequipa 04013, Peru; 5 Department of General, Visceral and Vascular Surgery, University Hospital Jena, Jena 07743, Germany

**Keywords:** radiation, therapy, radical, surgery, prostate, cancer, adjuvant, salvage

## Abstract

Radiation therapy plays a key role in the treatment of prostate cancer on its own. For higher risk diseases, the risk of recurrence following single modality therapy increases and a combination of treatment modalities may be necessary to achieve optimal results. We review clinical outcomes of adjuvant and salvage radiotherapy following radical prostatectomy, including disease-free survival, cancer-specific survival and overall survival. We also discuss when best to intervene with post-prostatectomy radiotherapy.

## Introduction

Prostate cancer is the most prevalent malignant disease in men worldwide. According to GLOBOCAN 2020, almost 5 million cases have been diagnosed in the last 5 years [1]. The incidence is estimated near 1.4 million new cases per year and the mortality rate is calculated at 375,000 cases each year around the globe. According to Siegel *et al* [27], only in the US, 288,300 new cases of prostate cancer will be reported in 2023, and 34,700 death related to this malignancy will occur in the same year. The probability of developing prostate cancer among men between 50 and 59 years old, is 1.8%, between 60 and 69 years old, is 5.2% and 70 years old and older, 9.2%. The overall probability of developing prostate cancer from birth to death is 12.6%, which signifies that 1 in 8 men will develop the disease in time.

Radiation therapy to the prostate gland is a highly effective treatment option for most men with localised prostate cancer. The evidence has shown that radiation therapy in its many forms of delivery can treat effectively prostate cancer and cure this disease [2]. Newer radiation techniques have been developed to treat with more precision the prostate gland, sparing organs nearby and reducing toxicities in the surrounding structures. These include brachytherapy, intensity-modulated radiotherapy and stereotactic body radiotherapy.

The use of radiation therapy after radical prostatectomy is used in two different scenarios. Adjuvant radiation therapy (ART) refers to radiation delivered shortly after surgery (usually within 6 months) to patients considered at high risk of future recurrence. The rationale for using ART can be summarised in four points: positive surgical margins, high-risk pathological features, preventing metastasis and improving local control of the malignancy [3–5].

Salvage radiation therapy (SRT) refers to treatment delivered for locally recurrent disease, usually identified by rising prostate-specific antigen (PSA) levels with or without evidence of clinical local recurrence.

We will describe both methods of radiation therapy after radical surgery, the evidence supporting both kinds of treatment, and a glimpse into the future of the management of high-risk localised and locally advanced prostate cancer.

## PSA persistence/recurrence and features of the final specimen after radical surgery

Usually in patients diagnosed with high-risk local or locally advanced prostate cancer that underwent radical surgery, a particular behaviour of PSA, which is used for monitoring the disease history, is expected. PSA should fall to undetectable levels following surgery.

PSA persistence means that the value of PSA after 4–8 weeks of the surgery according to most of the trials devoted to the matter, is higher than 0.1 ng/ml [2]. This generally is taken to indicate that some disease still remains, although on some occasions stable low values following prostatectomy may arise from residual benign prostate tissue left at the surgical margin.

PSA recurrence refers to a rising PSA value after initial treatment, either with surgery or radiation [2]. In the case of those patients that underwent surgery, if the PSA value is higher than 0.2 ng/ml during follow-up, a biochemical recurrence is defined. When the patient had radiation therapy as treatment with the intention to cure, the Phoenix criteria are used, and the value observed during follow-up is 2 ng/ml plus the nadir obtained from the various PSA values after radical treatment.

If the patient had a radical prostatectomy, valuable prognostic information is obtained from the pathologic specimen. If the final specimen demonstrates positive surgical margins, pathologic stage greater than T3a, positive nodal status and/or pathologic International Society of Urological Pathology (ISUP) grade more than 3, a poor prognosis is expected for the patient, and the physician in charge should evaluate multimodal treatment.

There have been established two scenarios of radiation therapy after radical surgery once we know all these features: ART and SRT. Both are discussed and compared here.

## Adjuvant radiation therapy

ART in the setting of prostate cancer already treated with radical prostatectomy involves delivering radiation treatment to target presumed microscopic disease before PSA failure has occurred. The goal of adjuvant treatment is to eradicate subclinical disease in order to delay or prevent further biochemical and clinical recurrence. It is primarily offered to those deemed at high risk of recurrence following surgery by virtue of having locally advanced or margin-positive disease.

The value of adjuvant radiotherapy following prostatectomy is well established and supported by several large randomised trials. These trials, in general, included patients considered at high risk of failure due to adverse pathology and randomised them to immediate adjuvant radiotherapy, or a watch-and-wait approach.

There are four randomised clinical trials that have been largely discussed in the world of uro-oncology. They will be summarised in Table 1.

### SWOG 8794

In 2008, Thompson *et al* [3] published an important randomised controlled trial. 425 patients were randomised 1:1 between ART, delivered in 60–64 Gy (214 subjects) and observation (211 subjects) after radical surgery. Those subjects eligible for the trial were diagnosed with locally advanced prostate cancer pT3N0M0. The main objective was metastasis-free survival, and the median follow-up was 12.7 years for the radiation arm and 12.5 years for the observation arm.

Of the 211 patients randomised for observation, 114 reached the event (metastasis development) and of 214 patients randomised for ART, 93 reached the event (Hazard ratio (HR) 0.71 with 95% CI 0.54–0.94, *p* = 0.016). Overall survival was also improved in the radiation arm, with a 10-year estimate of overall survival of 74% and 66% in the adjuvant and observation arms, respectively (*p* = 0.023). Median overall survival was increased from 13.3 to 15.5 years.

Researchers concluded that ART should be recommended over observation for men with pT3N0M0 prostate cancer after achieving positive results and, by default, the main objective of the trial. This improvement in outcome, however, came at a cost of greater toxicity, with an overall complication rate of 12% in the observation arm and 24% following adjuvant radiotherapy.

### EORTC 22911

In 2012, Bolla *et al* [4] published a seminal article. A 10-year follow-up study related to ART was presented. 1,005 patients aged 75 years or younger with a diagnosis of cT3-0 prostate cancer without treatment were recruited and randomised 1:1. External beam radiation therapy (60 Gy in a 6 weeks period of time) as an adjuvant treatment was employed in the experimental arm of this multicentre randomised clinical trial (502 participants). In the observation group (503 participants), a policy of wait-and-see was established. The main outcome was biochemical progression-free survival. After a median follow-up of 10.6 years, postoperative irradiation presented an improvement in biochemical progression-free survival compared with the controlled arm. 198 of 502 patients in the experimental arm versus 311 of 503 patients in the wait-and-see group (39.4% versus 61.8%, HR 0.49 (95% CI 0.41–0.59), *p* > 0.0001) reach the event for the survival analysis test. Although adjuvant treatment halved the risk of biochemical failure, this was not associated with an improvement in overall survival. Late adverse effects (any type of any grade) were more frequent in the postoperative irradiation group than in the wait-and-see group (10 years cumulative incidence 70.8% (95% CI 66.6%–75.0%) versus 59.7% (95% CI 55.3%–64.1%); *p* = 0.001).

After careful examination of the results, the authors concluded a real improvement could be seen in patients younger than 70 years old, with positive surgical margins but after 5 years of follow-up, the main outcome was not maintained. These facts promoted the design of more randomised clinical trials to establish the role of ART after radical surgery.

### ARO 96-02

In 2014, Wiegel *et al* [5] presented a new randomised controlled trial that started in 2009 and after, again, 10 years of follow-up, data was ready and mature to be shown to the academic world. 307 patients were randomised for three-dimensional conformal ART with 60 Gy in 6 weeks (148 participants) or the wait-and-see policy (159 participants). The main outcome was biochemical progression-free survival. The median follow-up was between 111 and 113 months for each group, respectively. A progression-free survival of 56% was observed in the experimental arm and for the control group, 35% (HR 0.51 95% CI 0.37–0.70, *p* < 0.0001). Few grade 2 or 3 events were reported and no grade 4 events occurred.

The authors explained that even if metastasis-free survival or overall survival were not statistically different, for patients with pT3 prostate cancer, ART improved the main outcome with an HR of 0.51. In patients with poor prognosis features, ART was an alternative with great expectations for the future.

### FinnProstate group trial

In 2019, Hackman *et al* [6] published a new piece of evidence of the highest level, a randomised multicentre open-label parallel-group trial, recruiting 250 patients with pT2 and positive margins or pT3a, pN0, M0 prostate cancer and performing randomization 1:1 between ART (66.6 Gy–126 participants) and observation (124 participants). The main outcome was biochemical progression-free survival. The median follow-up was 9.3 years in the adjuvant group and 8.6 years in the observation group.

The 10 years biochemical failure-free survival was 82% in the experimental arm and 61% in the observation group (HR 0.26 95% CI 0.14–0.48, *p* < 0.001). In the adjuvant group, 56% experienced grade 3 adverse events, versus 40% in the observation group (*p* = 0.016). Only one grade 4 adverse event occurred in the adjuvant group.

Related to overall survival, results between the two groups were not significantly different, but because the primary outcome was met, the FinnProstate group concluded that ART following radical prostatectomy prolongs biochemical-free survival with patients with positive margins or extracapsular extension.

## Salvage radiation therapy

SRT in the setting of prostate cancer already treated with radical surgery is a form a radiotherapy that, like its name, plays a role when signs of the return of the disease are present (i.e., PSA recurrence). Salvage rather than adjuvant treatment has the advantage of treating only patients who need it, and of avoiding treating many patients who would never have recurred. Most recurrences tend to occur in the prostate bed, with a rising PSA as the first indication.

Unfortunately, current imaging is unlikely to be able to detect the location of disease at an early enough stage due to limited sensitivity, even using molecular imaging. While selective imaging is recommended [7, 8], in most clinical scenarios salvage radiotherapy is recommended to be delivered before the site of recurrence is identifiable on imaging.

There is no high-quality evidence supporting an early salvage approach. Two randomised clinical trials further explored the use of SRT combined with short-term androgen deprivation therapy. One phase three randomised controlled trial had more arms evaluating SRT alone, combined with short-term androgen deprivation therapy and SRT to the prostatic bed and the lymph nodes field combined with, again, short-term androgen deprivation therapy. All these studies will be reviewed and summarised in Table 2.

### RTOG 9601

In 2017, Shipley *et al* [9] published a double-blind placebo-controlled trial exploring SRT (64.8 Gy in 36 daily fractions) with or without androgen deprivation therapy combined. Patients were randomised between salvage radiotherapy and 24 months of bicalutamide 150 mg daily, or salvage therapy alone.

Bicalutamide is an anti-androgen receptor drug of the first generation and it is taken orally. The main objective was, interestingly, overall survival. The median follow-up was 13 years for those patients who reached the event.

384 patients were randomised for salvage treatment combined with androgen deprivation therapy and the actuarial rate of overall survival at 12 years was 76.3%, as compared with 71.3% in the group without bicalutamide with 376 patients (HR for death, 0.77; 95% CI 0.59 to 0.99; two-sided *p* = 0.04). The 12-year incidence of death from prostate cancer was 5.8% in the bicalutamide group, as compared with 13.4% in the SRT alone group (HR 0.49, 95% CI, 0.32–0.74, *p* < 0.001). A similar incidence of late adverse events associated with radiation therapy was reported in the two groups. However, gynecomastia was recorded in 69.7% of the patients in the bicalutamide group, as compared with 10.9% of those in the placebo group (*p* < 0.001).

Researchers concluded that combined treatment should be advised for men with prostate cancer and biochemical recurrence, who underwent radical prostatectomy, given the results of this trial, although bicalutamide as monotherapy is no longer considered the optimal form of ADT.

### GETUG-AFU 16

In 2019, Carrie *et al* [10] presented a new open-label multicentre phase 3 randomised controlled trial exploring SRT (66 Gy in 33 fractions) with or without androgen deprivation therapy combined, using in this case, goserelin 10.8 mg for a period of 6 months. Goserelin is a LHRH analogue drug and it comes in a subcutaneous injection. 743 patients were randomised 1:1 between salvage radiotherapy and 6 months of goserelin (369 participants), or salvage therapy alone (374 participants). The main objective was progression-free survival in the intention-to-treat population. The median follow-up was 112 months.

The patients who were randomised for salvage treatment combined with androgen deprivation therapy presented a 120-month progression-free survival of 64% (95% CI 58%–69%) and 49% (95% CI 43–54) for the patients treated with radiotherapy alone (HR 0.54, 95% CI 0.43–0.68, stratified log-rank test *p* < 0.0001). Adjuvant goserelin improved the metastases-free survival with an HR of 0.73, *p* = .03339. No treatment-related deaths occurred.

The main conclusion was that the addition of 6-month of ADT reduced the risk of metastases compared with SRT alone.

### The SPPORT trial

In May 2022, Pollack *et al* [11] published an international multicentre randomised controlled trial that recruited 1,792 patients. It explored SRT for the prostatic bed (64.8–70.2 Gy at 1.8 Gy daily) and the pelvic lymph nodes field (45 Gy at 1.8 Gy per fraction, and then a volume reduction made to the planning target volume for the remaining 19.8–25.2 Gy) with androgen deprivation therapy combined, using a LHRH analogue (for a period of 4–6 months) and an anti-androgen receptor drug of first generation (used until the last day of radiation therapy and to last 4 months), and this treatment regimen was considered the experimental arm with all the interventions (EXP1, 598 participants). A second arm only received SRT for the prostatic bed and androgen deprivation therapy in the scheme previously presented (EXP2, 602 participants), and finally a third arm of subjects, who only received salvage radiotherapy to the prostatic bed without androgen deprivation therapy (CTRL, 592 participants). The main objective was freedom from progression, in which progression was defined as biochemical recurrence, clinical failure (local, regional, or distant), or death from any cause. The median follow-up was 8.2 years.

The patients who were randomised for salvage treatment to the prostatic bed and the lymph nodes field combined with androgen deprivation therapy reported a 5-year freedom from progression rate of 87.4% (95% CI 84.7–90.2). For the salvage therapy only to the prostatic bed and androgen deprivation therapy, participants reported a 5-year freedom from progression rate of 81.3% (95% CI 78.0–84.6) and 70.9% (95% CI 67–74.9) was the 5-year freedom from progression for participants treated with radiotherapy alone. According to the final analysis related to the main endpoint, freedom from progression rate was superior from EXP2 when compared to EXP1 and CTRL. It is important to comment that late toxicity (>3 months after radiotherapy) did not differ significantly between the groups, apart from more late grade 2 or worse blood or bone marrow events in EXP1 versus EXP2 (one-sided* p* = 0.006) attributable to the addition of pelvic lymph nodes radiotherapy in this group.

The research group concluded that salvage radiotherapy to the prostatic bed and the lymph nodes field combined with a short-term androgen deprivation therapy offered a benefit in progression-free survival and they presented evidence of the highest quality, establishing a potential standard of care.

## Comparison of ART and SRT

The evidence comparing both kinds of approaches has been recently established through three seminal randomised clinical trials and an important meta-analysis. This helps inform clinicians in the field to determine optimal timing of radiation delivery following prostatectomy in order to maximize clinical outcomes while minimizing toxicity and adverse impact on quality of life. The trials will be reviewed in the following lines and summarised in Table 3. A comment related to the meta-analysis will also be provided.

### TROG 08.03/ANZUP RAVES

In September 2020, Kneebone *et al* [12] published a non-inferiority multicentre phase 3 randomised controlled trial of the TROG/ANZUP group exploring early SRT versus ART after radical prostatectomy. In both groups, radiation therapy was delivered achieving 64 Gy in 32 fractions. The intended study population should have been 470 patients, but the research group only recruited 333 subjects. Eligible patients had pT3 disease, with or without positive margins, and PSA <0.1 ng/ml. The main objective was biochemical progression-free survival. Half of the patients randomised to the salvage arm developed biochemical failure and underwent salvage radiotherapy. The median follow-up was 6.5 years for those patients who reached the event.

167 patients were randomised for early salvage treatment (when PSA rose to 0.2 ng/ml) and the 5-year freedom from biochemical progression was 87% (95% CI 82–93), compared with 86% (95% CI 81–92) in the group from 166 subjects who received adjuvant radiotherapy (HR 1.12, 95% CI, 0.65–1.9, *p* = 0.15 (non-inferiority)). The grade 2 or worse gastrointestinal toxicity rate was similar between the salvage radiotherapy group (16 (10%)) and the adjuvant radiotherapy group (24 (14%)).

The researchers concluded that early salvage treatment resulted in a similar biochemical progression rate to immediate adjuvant treatment, sparing half of patients the potential morbidity of radiation.

### GETUG-AFU 17

In October 2020, Sargos *et al* [13] published a second open-label multicentre phase 3 randomised controlled trial from the European group exploring early SRT versus ART after radical prostatectomy (in both cases, they used 66 Gy in 33 fractions), but they allowed the combination with androgen deprivation therapy (LHRH analogue, in this case, triptorelin for 6 months). The intended study population should have been 718 patients, but the research group only recruited 424 subjects. Eligible patients had pT3/pT4 disease with a positive margin and PSA <0.1 ng/ml. The main objective was biochemical progression-free survival, and the median follow-up was 6.25 years for those patients who reached the event.

212 patients were randomised for salvage treatment (when PSA rose to 0.2 ng/ml) and the 5-year freedom from biochemical progression was 90% (95% CI 85–94), compared with 92% (95% CI 86–95) in the group of 212 subjects who received adjuvant radiotherapy (HR 0.81, 95% CI, 0.48–1.36, *p* = 0.42). There was notably higher late grade 2 and 3 GU toxicity in the adjuvant arm (27% versus 7%).

Although lacking statistical power to prove non-inferiority, the results were consistent with those of the RAVES study and adjuvant radiotherapy should not be the first option when biochemical recurrence was diagnosed in patients with prostate cancer who underwent radical prostatectomy.

### RADICALS-RT

In September 2020, Parker *et al* [14] published the largest multicentre randomised controlled trial exploring early SRT versus ART after radical prostatectomy. In both cases, the radiation therapy was equivalent to a non-randomised dose-fractionation schedule of either 66.0 Gy in 33 fractions or 52.5 Gy in 20 fractions. Participants could also receive pelvic lymph node radiotherapy at the discretion of the physician. The intended study population of 1,396 patients was reached. Eligibility criteria were broader than for the other two studies – patients with pT3 and/or positive margins, Gleson 7-–10 and pre-op PSA >10 were all eligible. Furthermore, SRT was administered at an earlier stage, when PSA rose to 0.1 ng/ml. The primary endpoint was biochemical progression-free survival, and the median follow-up was 4.9 years for those patients who reached the event.

699 patients were randomised for salvage treatment and the 5-year biochemical progression-free survival was 88% and 85% for those in the adjuvant radiotherapy group with 697 subjects (HR 1.10, 95% CI 0.81–1.49, *p* = 0·56). Urinary incontinence was worse at 1 year for those in the adjuvant radiotherapy group (mean score 4.8 versus 4.0; *p* = 0.0023). Grade 3–4 urethral stricture within 2 years was reported in 6% of individuals in the adjuvant radiotherapy group versus 4% in the salvage radiotherapy group (*p* = 0.020).

The RADICALS-RT research team concluded that a strategy of very early salvage was non-inferior to immediate adjuvant radiotherapy in this patient population.

### ARTISTIC

Vale *et al* [15] performed a pre-planned ARTISTIC meta-analysis of these three randomised trials. The research team redefined the event-free survival, as the time from randomization until the first evidence of either biochemical progression, clinical or radiological progression, initiation of a non-trial treatment death from prostate cancer or PSA level of at least 2 ng/ml at any time after randomization.

2,153 participants were gathered after applying elegibility criteria and only 3 clinical trials were available for analysis. The patients assigned to receive adjuvant radiotherapy were 1,075 and 1,078 received early salvage radiotherapy, of whom 421 (39.1%) had commenced treatment at the time of analysis. According to 270 events, the meta-analysis showed no evidence that event-free survival was improved with adjuvant radiotherapy compared with early salvage radiotherapy (HR 0.95, 95% CI 0.75–1.21; *p* = 0.7), with only a 1% point (95% CI −2–3) change in 5-year event-free survival (89% versus 88%). Results were consistent across trials (heterogeneity *p* = 0·18; I2 = 42%).

Vale *et al* [15] concluded that the systematic use of adjuvant radiotherapy following prostatectomy dose not improve recurrence-free survival compared with a policy of early salvage for men with localised or locally advanced prostate cancer.

Several trials are still ongoing, and the present evidence has opened the door for improving the data related to radiation therapy after radical surgery in prostate cancer. Table 4 will summarise some of the most important present studies registered in Clinicaltrials.gov related to this matter.

## Discussion

Nowadays, physicians can offer a vast variety of treatments in various disease scenarios, and overall survival rates are rising [2]. Therefore, if the patient is living longer, the quality of life should be as good as possible [23], and harm due to the chosen treatment should be avoided.

Radical surgery is an option in many malignancies where there is a solid tumour. Following the principles of surgical oncology, surgeons may perform a procedure and remove the primary tumour if negative margins can be achieved. In prostate cancer, radical prostatectomy is also an option, but it is often associated with positive margins or biochemical recurrence [24].

Radiation therapy on its own has proven to be a standard alternative treatment for curing prostate cancer. Experts and medical literature advise combining treatments to achieve long-standing remission and a solution for the disease when dealing with localised high-risk or locally advanced prostate cancer [2]. The present review stands the scenario of a patient who has already undergone radical surgery to cure the disease. However, as we have learned from the natural history of high-risk prostate cancer [25], it will recur unless we provide another form of therapy, which in this case is radiation therapy.

In that situation, post-prostatectomy radiotherapy, most of the time with androgen deprivation therapy, is often indicated. For patients with undetectable or very low PSA (<0.1 ng/ml) after surgery, close monitoring is an option, with early intervention with radiation treatment once biochemical failure is documented. For those with higher PSA levels, immediate radiation with adjuvant ADT should be considered.

Multi-modality assessment of patients with high-risk prostate cancer should be encouraged. The range of treatment options should be discussed, and those choosing to proceed with primary prostatectomy should be aware that there is a high possibility of needing subsequent radiotherapy and ADT if pathology is unfavourable or if a biochemical failure occurs.

Currently, there is no clear consensus about which kind of post-prostatectomy radiotherapy should be offered to patients. The latest body of evidence presented here points towards establishing that SRT should be the new standard of care after radical prostatectomy, as one clinical trial [14] and a meta-analysis [15] suggest. However, in an era when heterogeneity of features among subjects is the rule, the statement that one treatment fits all fails.

A recent publication by Tilki *et al* [16] analysed the data from the trials involved in the comparison between adjuvant radiotherapy and SRT, the ARTISTIC meta-analysis. After careful consideration of patients with adverse pathological features, adjuvant compared with early SRT was associated with significantly lower all-cause mortality risk among men with these features, whether pelvic lymph nodes were positive for disease (adjusted hazard ratio (AHR) = 0.66 (95%CI: 0.44–0.99); *p* = .04) or not (AHR = 0.33 (95%CI:0.13–0.85); *p* = 0.02).

Both types of radiation therapy have benefits and drawbacks. On one hand, adjuvant radiotherapy can achieve better results in terms of treatment, such as recurrence-free survival, but if the patient suffers from urinary incontinence, the problem can become permanent after radiation therapy [24]. On the other hand, SRT can spare the patient from unnecessary toxicity resulting from the combination therapy, but as a recent study has shown, if it's not given before a total PSA of 0.25 ng/ml, its efficacy won’t be adequate, and the patient won’t benefit from longer recurrence-free survival [26].

In the end, the decision-making process involving the next step in the treatment of such patients will have to consider the patient's full profile and the expertise of the physician in charge. The ideal solution would involve a multidisciplinary team that should discuss all cases in a particular institution.

## Conclusion

Radiation therapy after radical prostatectomy is the standard of care for patients with high-risk pathology or rising PSA value. There are two strategies in this modality: adjuvant radiotherapy and salvage radiotherapy. Both strategies are supported by high-quality evidence. Therefore, careful selection of patients should guide the therapeutic pathway for each subject because of heterogeneity and conditions the patient may present at the moment of making the decision. Finally, further trials should be launched in order to define better the treatment of high-risk prostate cancer.

## Author contributions

Roberto Paz-Manrique and Santiago Paz-Manrique made the contributions to the conception and design of the work. Roberto Paz-Manrique, Santiago Paz-Manrique and Gerard Morton made the first draft. Fernando Quiroa Vera, Andrés Espinoza Briones and Carlos Morante Deza critically reviewed the draft for intellectual content and made significant changes. All authors approved the final version of the manuscript.

## Conflicts of interest

Gerard Morton

Honoraria: Elekta

The other authors declare that they have no financial or non-financial conflicts of interest.

## Funding statement

No funding was required for this article.

## Figures and Tables

**Table 1.** Clinical trials related to ART.

Trial	Design	Population	Intervention	Control	Outcome	Results	Statistical significance
SWOG 8794	Phase 3	425	ART (*N* = 214)	Obs (*N* = 211)	MFS	HR = 0.71	*p* = 0.016
EORTC 22911	Phase 3	1,005	ART (*N* = 502)	Obs (*N* = 503)	BPFS	HR = 0.49	*p* < 0.0001
ARO 96-02	Phase 3	307	ART (*N* = 148)	Obs (*N* = 159)	BPFS	HR = 0.51	*p* < 0.0001
FinnProstate group trial	Phase 3	250	ART (*N* = 126)	Obs (*N* = 124)	BPFS	HR = 0.26	*p* < 0.001

**Table 2.** Clinical trials related to SRT.

Trial	Design	Population	Intervention	Control	Outcome	Results	Statistical significance
RTOG 9601	Phase 3	760	SRT + ADT (*N* = 384)	SRT alone (*N* = 376)	OS	HR = 0.77	*p* = 0.04
GETUG-AFU 16	Phase 3	743	SRT + ADT (*N* = 369)	SRT alone (*N* = 374)	PFS in the ITT Pop.	HR = 0.54	*p* < 0.0001
SPPORT trial	Phase 3	1,792	eSRT + PLNRT + ADT (*N* = 598) eSRT + ADT (*N* = 602)	eSRT (*N* = 592)	PFS	HR = 0.39 (between EXP1 and CTRL	*p* < 0.001

**Table 3.** Clinical trials related to the comparison between adjuvant and SRT.

Trial	Design	Population	Intervention	Control	Outcome	Results	Statistical significance
TROG 08.03/ANZUP RAVES	Phase 3	333	SRT (*N* = 167)	ART (*N* = 166)	BPFS	HR = 1.12	*p* = 0.15
GETUG-AFU 17	Phase 3	424	SRT (*N* = 212)	ART (*N* = 212)	BPFS	HR = 0.81	*p* = 0.42
RADICALS-RT	Phase 3	1,396	SRT (*N* = 699)	ART (*N* = 697)	BPFS	HR = 1.10	*p* = 0.056

**Table 4.** Ongoing trials registered in Clinicaltrials.gov.

Trial	Design	Population	Intervention	Comparison	Primary objective
NCT04134260 [17]	Phase 3	Prostate cancer CS I-IVa with radical surgery	SRT + ADT + Apalutamide	SRT + ADT	MFS
NCT01272050 [18]	Phase 3	Prostate cancer with radical surgery	SRT (70 Gy)	SRT (64 Gy)	Freedom from biochemical progression
SPPORT [11]	Phase 3	Prostate cancer with radical surgery	SRT (Ex.) + PLNRT + ADT SRT (Ex.) + ADT	SRT (Ex.) alone	Freedom from biochemical progression
NCT00667069 [19]	Phase 3	Prostate cancer with radical surgery	SRT + ADT	ART + ADT	Event free survival
NCT00860652 [20]	Phase 3	Prostate cancer with radical surgery	Early SRT	ART	Biochemical failure
NCT05067660 [21]	Pilot trial	Prostate cancer with radical surgery	Stereotactic Ablative SRT	Standard SRT	Undetectable serum PSA after SRT
NCT01868386 [22]	Phase I/II	Prostate cancer with radical surgery	Hypofractionated RT (various schemes)	-	To determine the shortest dose-fractionation schedule
